# Regulating Lithium Plating Behavior in Lithium‐Metal Batteries via Molten‐Lithium Processing With Inorganic Additives

**DOI:** 10.1002/smll.74228

**Published:** 2026-06-18

**Authors:** Chaerim Kim, Jinyeong Choi, Chaerin Jung, Jihyeon Kang, Hyeongbeen Kim, Pilgun Oh, Jin Hong Lee, Sung Hyun Kwon, Minjoon Park

**Affiliations:** ^1^ Department of Nano Fusion Technology Pusan National University Busan Republic of Korea; ^2^ Research Center of Energy Convergence Technology Pusan National University Busan Republic of Korea; ^3^ Eco‐friendly Battery Engineering Pusan National University Busan Republic of Korea; ^4^ Department of Smart Green Technology Engineering Pukyong National University Busan Republic of Korea; ^5^ School of Chemical Engineering Pusan National University Busan Republic of Korea; ^6^ Advanced Energy Major School of Transdisciplinary Engineering Pusan National University Busan Republic of Korea

**Keywords:** alloy, lithium metal anode, lithium metal battery, lithium, molten lithium, zinc fluoride

## Abstract

Lithium metal batteries (LMBs) are compelling candidates for next‐generation energy storage owing to the ultrahigh theoretical capacity (3,860 mAh g^−1^) and high energy density (400–500 Wh kg^−1^). However, uncontrollable dendrite growth, severe volume change, low Coulombic efficiency, and the poor lithiophilicity of Cu current collectors have impeded practical deployment. Here, we report a self‐assembled gradient interphase (SGI) produced in situ by reacting molten lithium with ZnF_2_. The SGI exhibits a self‐formed, vertically graded architecture. The SGI consists of a LiZn alloy sublayer that lowers nucleation overpotential and accelerates Li^+^ transport, and a LiF layer that offers high ionic conductivity and outstanding air stability for durable interfacial stability. The lower LiZn alloy layer promotes uniform lithium nucleation by providing a low diffusion barrier and strong interfacial affinity, while the upper LiF layer forms a stable, inorganic SEI that effectively suppresses dendrite growth. The symmetric cell equipped with the SGI Li–Zn–F interphase exhibited highly durable cycling, maintaining stable operation for 3000 h under 4 mA cm^−2^/16 mAh cm^−2^. This dual‐layer configuration, in which distinct functional roles are spatially separated within the interphase, offers an advantage not attainable with conventional single‐layer coatings.

## Graphical abstract

A SGI Li–Zn–F is formed by introducing ZnF_2_ into molten lithium, generating a LiZn alloy layer beneath a LiF‐rich upper layer. The lithiophilic LiZn alloy layer and robust LiF layer synergistically regulate Li^+^ flux and suppress dendritic growth, enabling uniform Li deposition and long‐term stable cycling in lithium metal batteries.

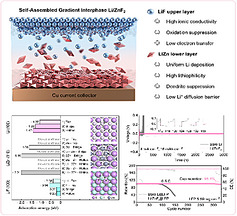

## 1 Introduction

With the rapid expansion of the global electric vehicle (EV) market, lithium‐ion batteries (LIBs), the core power source for EVs, have achieved remarkable technological advancements [1, 2]. However, the growing demand for batteries has intensified the need for higher energy density and improved efficiency. Graphite, the conventional anode in commercial LIBs, is intrinsically constrained by its limited specific capacity (372 mAh g^−1^) and moderate energy density, which hinder further improvements in energy output and vehicle range [3, 4]. To overcome these intrinsic limitations, lithium metal anodes (LMAs) have emerged as a promising alternative owing to their ultrahigh theoretical capacity and the lowest electrochemical potential among known anode materials [5, 6].

LMAs possess an ultrahigh theoretical specific capacity (3860 mAh g^−1^), a low redox potential (−3.04 V vs SHE), and a low density (0.534 g cm^−3^), making them highly attractive for next‐generation high‐energy batteries [7, 8, 9, 10, 11]. However, their practical application remains hindered by severe volume fluctuations during repeated Li plating/stripping. Continuous cycling leads to uneven Li deposition, where localized high‐current regions trigger the growth of needle‐like dendrites [12, 13]. These dendritic structures can penetrate the separator and induce internal short circuits, ultimately compromising the safety and lifetime of the cell [14, 15]. Intensive studies have focused on mitigating the volume change of lithium metal anodes and suppressing dendrite formation. The main strategies can be broadly categorized into three approaches: (1) electrolyte engineering [16, 17, 18, 19, 20], (2) current‐collector modification [21, 22], and (3) alloying [23]. Tailored electrolyte formulations can regulate local Li^+^ solvation structures and promote the formation of a robust solid electrolyte interphase (SEI) during cycling [24, 25, 26]. However, their long‐term stability at high current densities remains limited. The application of artificial interfacial layers such as ultrathin Si_3_N_4_ [27], Al_2_O_3_ [28], or polymer coatings [29], and structural modification of current collectors through 3D frameworks have effectively alleviated dendritic growth and homogenized local current distribution by acting as ionically conductive yet electronically insulating barriers [30, 31, 32, 33]. Despite these advances, such approaches often require complex synthesis routes and suffer from uncontrolled, off‐site Li deposition. Alloying strategies have long been explored because lithium readily reacts with specific metals via molten processing or electrodeposition to form stable alloys. In particular, the formation of lithiophilic alloys through the addition of a single metal has demonstrated improved interfacial wettability and enhanced cycling stability [34, 35, 36]. Nevertheless, this single‐metal approach primarily enhances Li affinity without resolving the intrinsic instability of the SEI, thereby limiting the overall interfacial durability of lithium metal anodes.

To achieve a stable and practical lithium metal anode, two inherently conflicting requirements must be simultaneously satisfied. First, the current collector must enable rapid and homogeneous lithium nucleation to avoid localized current concentration [37, 38, 39, 40]. Second, a chemically stable and ionically conductive interfacial layer is required to preserve long‐term SEI integrity against continuous electrolyte exposure [41, 42, 43]. Conventional single‐component or compositionally uniform interphases fail to decouple these dual functions, typically exhibiting either fast nucleation with limited interfacial stability or strong protection accompanied by sluggish ion transport. Consequently, a new interfacial design paradigm is needed, one that integrates spatially separated nucleation and protection functionalities within a single, unified structure.

Here, we report a self‐assembled gradient interphase (SGI) that spontaneously segregates into a LiZn lower layer adjacent to the copper current collector and a LiF‐rich upper layer facing the electrolyte. The LiZn alloy can provide a low diffusion barrier and abundant lithiophilic nucleation sites, while the LiF layer offers high ionic conductivity and chemical robustness. This vertically graded structure effectively decouples nucleation kinetics from interfacial stabilization, enabling dendrite‐free Li plating/stripping with minimal hysteresis for 3000 h under high current density (4 mA cm^−2^, 16 mAh cm^−2^). In full‐cell tests with LFP cathodes, the SGI anode exhibits stable capacity retention of approximately 98.4% over 330 cycles at 0.5 C, further validating its practicality through pouch‐cell fabrication. Notably, the SGI is realized via a simple dip‐coating and molten‐reaction process, eliminating the need for complex deposition methods such as chemical vapor deposition and ensuring compatibility with roll‐to‐roll electrode manufacturing. This dual advantage establishes the SGI as a viable platform concept that connects interfacial design principles with scalable manufacturing routes for practical lithium metal batteries.

## 2 Results and Discussion

### 2.1 The Mechanism and Fabrication of Li/ZnF_2_ Anode

**FIGURE 1:**
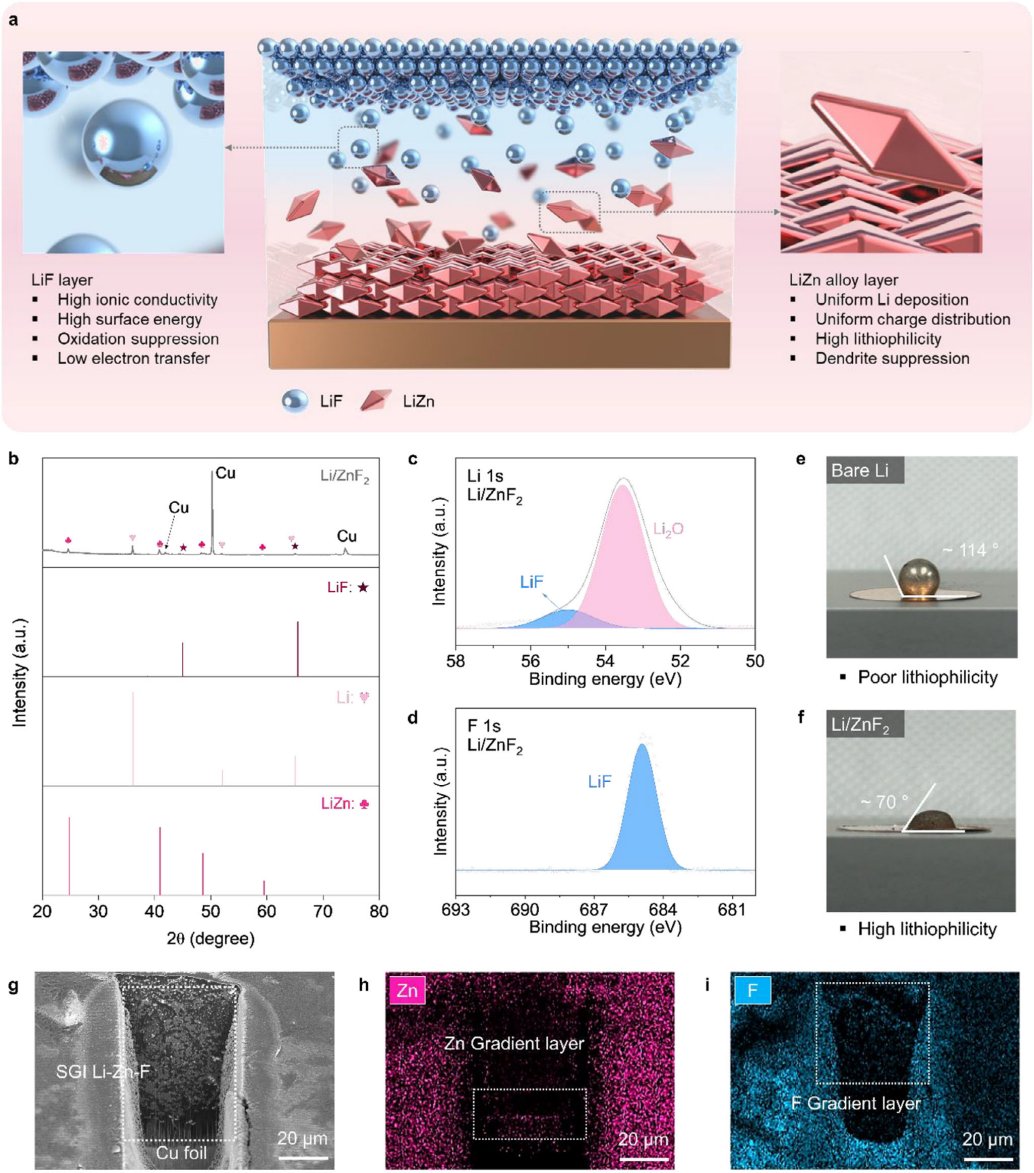
(a) Schematic illustration of the synthesis process of the Li/ZnF_2_ composite anode. (b) XRD patterns of the Li/ZnF_2_ electrode. XPS patterns of Li/ZnF_2_, (c) Li 1s, (d) F 1s. The digital images of contact angles of the (e) Bare Li and (f) Li/ZnF_2_. (g) FIB‐SEM images of the cross‐section of the Li/ZnF_2_ electrode and EDS mapping image of (h) Zn and (i) F atoms.

We formed an SGI consisting of a LiF‐rich upper layer and a LiZn alloy lower layer through an in situ reaction between molten lithium and Li/ZnF_2_. As shown in Figure 1a, the behavior of the Li/ZnF_2_ electrode is governed by the combined contributions of the LiZn alloy phase and the LiF‐rich layer. The high ionic conductivity and chemical robustness of LiF provide a stable foundation for rapid lithium nucleation, while the lithiophilic LiZn alloy offers favorable nucleation sites that promote preferential lithium plating and facilitate uniform lithium deposition. The presence of zinc and fluorine was confirmed in the scanning electron microscopy (SEM) and energy‐dispersive x‐ray spectroscopy (EDS) mapping of the ZnF_2_ powder used in the experiment (Figure S1). Figure S2 presents a schematic of the Li/ZnF_2_ electrode fabrication procedure. Informed by earlier reports on composite lithium anodes produced through mechanical folding and rolling of lithium foil with functional powders, we implemented a similar pretreatment strategy [44, 45, 46]. ZnF_2_ powder was first combined with lithium metal foil at a mass ratio of 20:1. The foil and powder mixture was then repeatedly folded and rolled, yielding a uniform lithium composite. This composite was subsequently heated to 280°C inside an inert glovebox environment and thoroughly stirred, forming a fluid, gray‐toned molten lithium mixture. Finally, the Li/ZnF_2_ electrode was obtained by dip‐coating the molten composite onto Cu foil.

Subsequently, x‐ray diffraction (XRD) analysis was performed to confirm the alloy and LiF peaks in the Li/ZnF_2_ electrode (Figure 1b; Figure S3). The main XRD peaks confirmed the formation of LiZn alloy, inorganic LiF, and metallic Li on the substrate. The peaks of the XRD analysis were identified as metallic Li, Cu alloy LiZn, and inorganic LiF. After reacting with lithium, the main peaks of LiZn alloy were confirmed to be located at 24.82°, 41.08°, and 48.59°, respectively, along the (111), (220), and (311) planes, while LiF was confirmed to be located at 40.99° and 65.5°. On the other hand, the XRD peaks of Bare Li only showed metallic Li and Cu (Figure S4). This result confirmed that LiZn and LiF were produced through the interfacial conversion reaction between ZnF_2_ and Li, following the reaction. The coexistence of LiF and LiZn phases confirms the formation of a vertically stratified SGI composed of an upper LiF layer and a lower LiZn alloy layer.

X‐ray photoelectron spectroscopy (XPS) further verified the interfacial chemistry. In the F 1s spectrum, the dominant peak at 685 eV is attributed to LiF [47], indicating the formation of a fluorine‐rich interphase. LiF(52.0 eV) [48, 49] was also observed in the Li 1s spectrum, indicating that the electrode surface consists of a LiF surface. The observed Li_2_O is believed to have originated from the oxidation reaction of lithium and oxygen during the sample transfer process. Enhancing the interfacial contact between lithium and Cu foil is important for the production of highly stable lithium metal batteries because it ensures uniform distribution of lithium on the Cu surface, thereby suppressing side reactions occurring during Li plating/stripping [6, 50]. Figure 1g,h shows that Bare Li exhibited a contact angle of 114° on the copper foil, indicating poor lithiophilicity between Cu and Li. In contrast, the Li/ZnF_2_ electrode exhibited a significantly reduced contact angle of 70°, reflecting enhanced lithium affinity. This improvement was attributed to the SGI Li–Zn–F, which enhanced the wettability of molten lithium and reduced the interfacial surface tension with the Cu foil. As a result, it can be uniformly spread on the surface of the copper foil and promote stable nucleation during the Li plating/stripping process.

Focused ion beam (FIB) milling was employed to resolve the microstructure of the Li/ZnF_2_ electrode. As shown in Figure 1i, the interior of the Li/ZnF_2_ electrode exhibits a compact nanoscale architecture arising from the formation of SGI Li–Zn–F. This unique hierarchical alloyed structure provides lithiophilic sites and a chemically robust framework, thereby mitigating parasitic side reactions and enhancing the overall electrochemical performance of the electrode. In addition, Figure 1h,i shows the energy‐dispersive x‐ray spectroscopy (EDS) mapping of Li/ZnF_2_, which showed that an abundant F element was accumulated at the top of the electrode and Zn element was distributed relatively at the bottom. Simply put, this structure showed a SGI Li–Zn–F, consisting of a LiF‐rich layer on top and LiZn on the bottom. In addition, the structure of the self‐assembled gradient interface could be observed via SEM‐EDS in Figure S5. Within the self‐assembled gradient interphase, Zn mapping in the form of nanodots was confirmed to be concentrated in the region closest to the Cu foil. In contrast, F was found to be enriched in the upper region, farther from the Cu foil, indicating a distinctly different spatial distribution compared to that of Zn. The clear spatial separation between Zn and F confirms that the self‐assembled gradient interphase is stratified, with LiZn concentrated in the bottom layer adjacent to the Cu foil and LiF enriched in the upper layer. LiF provides a stabilized interface due to its high ionic conductivity and chemical stability [51, 52, 53, 54, 55], while LiZn can offer low interfacial energy and abundant lithium affinity nucleation sites [56, 57, 58, 59]. The coexistence of these two phases allows them to play complementary roles. The SGI Li–Zn–F structure formed throughout the electrode effectively suppresses lithium dendrite growth during the Li plating/stripping process, resulting in a uniform electrode surface.

### 2.2 Interfacial Formation Mechanism of SGI Li–Zn–F

**FIGURE 2:**
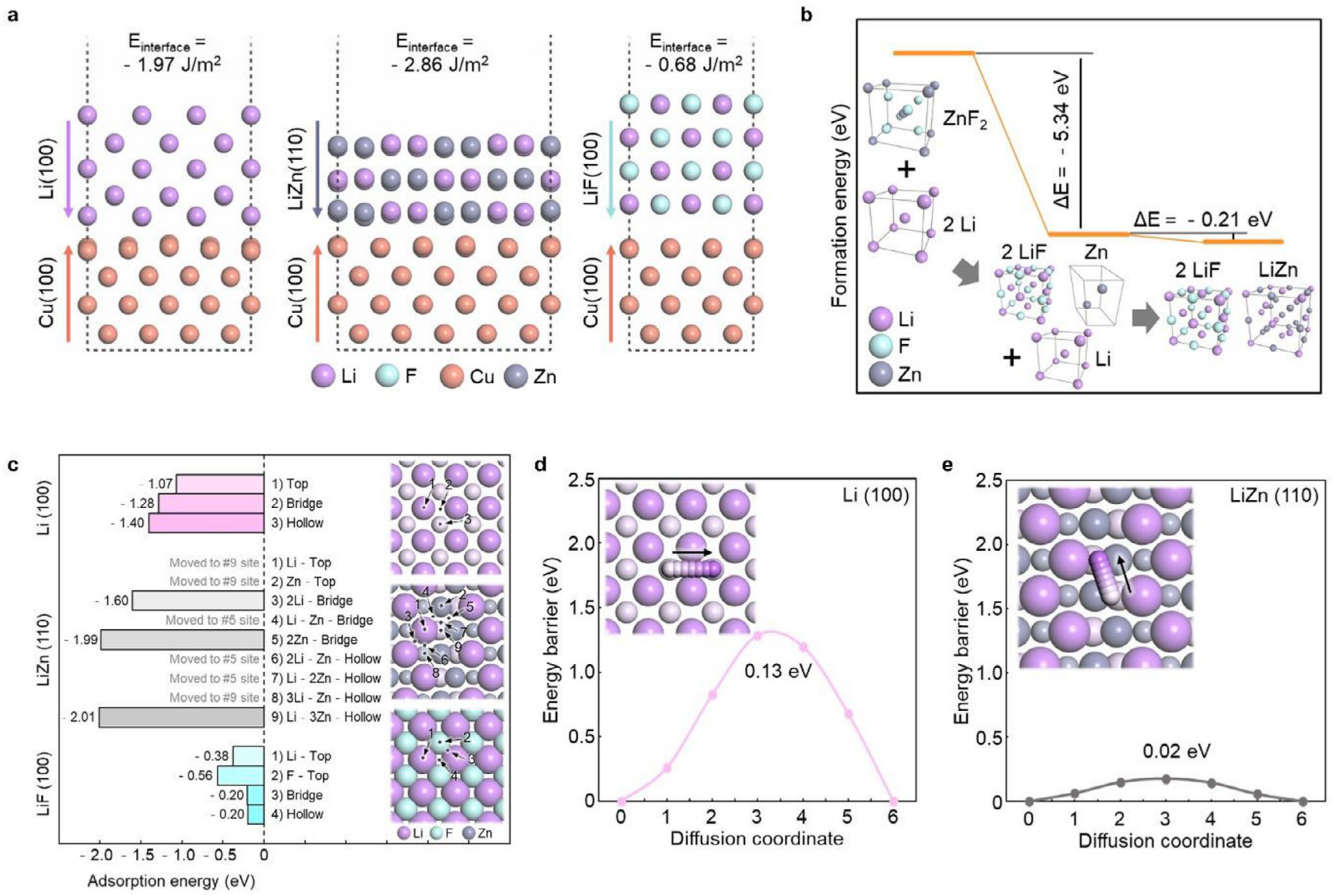
(a) Interface energies of Cu(100)|Li(100), Cu(100)|LiZn(110), and Cu(100)|LiF(100). (b) Schematic illustration of the formation energy from the reaction between ZnF_2_ and Li. (c) Li adsorption energies on Li(100), LiZn(110), and LiF(100) surfaces. The diffusion barriers of Li along (d) Li(100) and (e) LiZn(110) surfaces.

Density functional theory (DFT) calculations were conducted to elucidate the formation behavior of LiZn and LiF on Cu foil. As demonstrated in Figure 2a, the interfacial energies of Li, LiZn, and LiF on Cu were calculated, where more negative values indicate stronger interfacial adhesion and higher thermodynamic stability. The interfaces Cu(100)|Li(100) and Cu(100)|LiZn(110) yielded values of −1.97 and −2.86 J m^−2^, respectively, indicating that LiZn exhibits stronger chemical bonding and improved wettability with Cu compared to metallic Li. Conversely, the Cu(100)|LiF(100) interface exhibited a higher energy of −0.68 J m^−2^, suggesting weaker adhesion to Cu. These results indicate that LiZn preferentially forms at the Cu interface as the lower layer, while LiF, with weaker interfacial affinity, segregates toward the top region adjacent to the electrolyte. As shown in Figure S6, the adsorption energies of LiF(100), Li(100), and LiZn(110) surfaces were calculated to evaluate the interfacial interactions within the gradient interphase structure. The interfacial energy of Li(100)|LiZn(110) was determined to be – 0.45 J m^−2^, which is more negative than that of LiF(100)|Li(100) (− 0.29 J m^−2^), indicating that the LiF interface forms a stronger chemical bond with LiZn than with metallic Li. This difference in interfacial energy provides a thermodynamic driving force for the preferential formation of LiZn at the lower layer in direct contact with Li, while LiF is positioned at the upper layer, thereby establishing the theoretical basis for the observed gradient structure. Furthermore, this robust interfacial interaction effectively prevents delamination during Li plating/stripping and enhances mechanical integrity by maintaining stable interfacial contact. As illustrated in Figure 2b, the alloying reaction proceeds according to the following conversion pathway Equations (1) and (2).

(1) ZnF2+2Li→2LiF+Zn+Li,ΔE=−5.34Ev

(2) LiF+Zn+Li→LiZn+2LiF,ΔE=−0.21eV

The spontaneous formation of LiZn and LiF phases, occurring without the requirement for external energy input, resulted in thermodynamically stable products. These stable products suppressed interfacial corrosion at the electrolyte interface and facilitated uniform Li^+^ diffusion across the electrode [60, 61]. Consequently, the LiF and LiZn phases generated through the alloying reaction contributed to enhanced interfacial robustness, effectively preventing delamination or collapse of the electrolyte interface during repeated Li plating/stripping cycles. The strong adsorption energy and low diffusion barriers at the substrate are critical for achieving uniform Li deposition and dissolution. Li atoms that remain stably adsorbed and migrate rapidly across the surface promote homogeneous nucleation and growth. The possible adsorption sites on the Li(100), LiZn(110), and LiF(100) surfaces are illustrated in Figure S7–S9. As demonstrated in Figure 2c, the Li adsorption energy on the Li(100) surface was calculated to be −1.40 eV, whereas LiZn(110) exhibited a lower adsorption energy of −2.01 eV, indicating that Li^+^ preferentially nucleates on the LiZn alloy surface. The LiF(100) plane showed an adsorption energy of −0.56 eV, which is weaker than that of Li(100), indicating its poor affinity toward lithium. As a result, the SGI Li–Zn–F adopted a vertically stratified configuration, with LiF distributed at the top and LiZn at the bottom. The alloy component in the lower layer was strongly anchored to the Cu current collector, while the halide‐based LiF, which is relatively light and less compatible with Cu, migrated toward the upper region. Positioned adjacent to the electrolyte, the LiF layer acted as a dendrite‐suppressing barrier during Li plating and stripping.

As shown in Figure 2d,e, the Li^+^ diffusion barrier was calculated to elucidate transport behavior across the interfaces. The Li(100) surface exhibited an energy barrier of 0.13 eV, whereas LiZn(110) showed a markedly lower value of 0.02 eV, less than half that of Li(100). This exceptionally low diffusion barrier on LiZn facilitates rapid Li^+^ transport, contributing to homogeneous deposition and enhanced interfacial kinetics in the SGI‐structured electrode. This behavior is attributable to the low diffusion barrier of LiZn, which facilitated more efficient Li^+^ transport in comparison with unmodified Li and improved the wettability between lithium and the Cu current collector. During the dip‐coating process of molten lithium, LiZn and LiF phases were formed in situ through alloying reactions and subsequently segregated vertically according to their interfacial affinities. Owing to its low adsorption energy and diffusion barrier, LiZn exhibited strong adhesion to the Cu substrate, forming the lower layer, while the less adherent LiF phase migrated upward to constitute the upper layer. This vertically aligned configuration established a well‐defined elemental gradient, in which LiF, positioned closest to the electrolyte, facilitated Li^+^ conduction and served as a protective barrier against dendritic propagation. Meanwhile, the lithiophilic LiZn alloy at the bottom minimized interfacial resistance with Cu and enabled uniform lithium deposition during cycling.

### 2.3 Symmetric Cell Performance of Li/ZnF_2_ Anode

Symmetric cell tests were conducted to evaluate the cycling stability of Li/ZnF_2_ during repeated Li plating/stripping. Prior to assembly, the effect of ZnF_2_ loading was optimized (Figure S10). Li/ZnF_2_ composites were prepared with different mass ratios of 10, 20, and 30, and their performance was evaluated at 1 mA cm^−2^ and 1 mAh cm^−2^. Among them, the 20:1 composition exhibited the lowest nucleation overpotential and stabilized most rapidly, maintaining the smallest voltage polarization. Electrochemical impedance spectra (EIS) showed a similar trend, with Li/ZnF_2_ (20:1) displaying the lowest interfacial resistance, followed by 30:1 and 10:1 (Figure S11). Consequently, Li/ZnF_2_ (20:1) was selected for subsequent experiments because of its superior electrochemical stability and low resistance.

**FIGURE 3:**
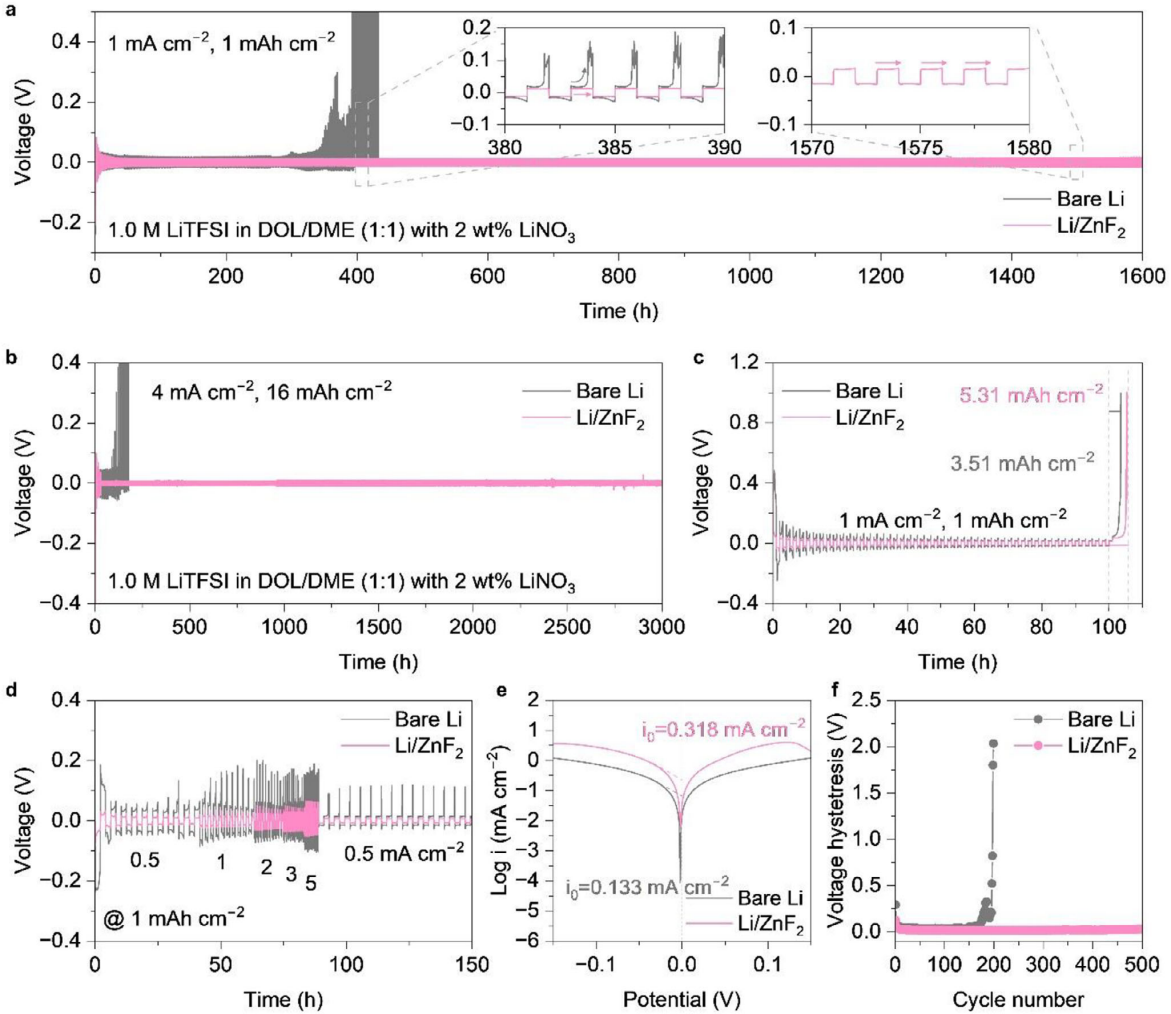
Galvanostatic plating and stripping of the symmetric cells voltage profiles at (a) 1 mA cm^−2^/1 mAh cm^−2^ and (b) 4 mA cm^−2^/16mAh cm^−2^ with Bare Li and Li/ZnF_2_. (c) The ACE of Bare Li and Li/ZnF_2_ for 50 cycles at 1 mA cm^−2^/1 mA h cm^−2^. (d) Rate performance of the symmetric cells with Bare Li and Li/ZnF_2_ at the different current densities from 0.5 to 5 mA cm^−2^. (e) Tafel plots of Bare Li and Li/ZnF_2_ were measured between −0.15 and 0.15 V at a scan rate of 1 mV s^−1^. (f) The corresponding voltage hysteresis with Bare Li and Li/ZnF_2_ at 1 mA cm^−2^/1 mA h cm^−2^.

At 1 mA cm^−2^ and 1 mAh cm^−2^, Bare Li exhibited increasing overpotential after 230 h and short‐circuited after 400 h once the voltage exceeded 400 mV. In contrast, Li/ZnF_2_ maintained a stable cycling performance after 1600 h with low voltage polarization (Figure 3a). Under more demanding conditions of 4 mA cm^−2^ and 16 mAh cm^−2^, Li/ZnF_2_ remained below 10 mV for 3000 h, whereas Bare Li exceeded 400 mV after 200 h because of unstable electrolyte/electrode interfaces (Figure 3b; Figure S12). These results confirm that the SGI Li–Zn–F provides outstanding cycling stability even under high‐current‐density and high‐capacity conditions.

Average Coulombic efficiency (ACE) measurements further demonstrated the uniform plating/stripping behavior of Li/ZnF_2_ (Figure 3c). Li/ZnF_2_ delivered a high Coulombic efficiency (96.4%) compared with Bare Li (94.7%), attributable to its larger inventory of activatable lithium (≈5.07 mAh cm^−2^) and homogeneous Li deposition and dissolution after 50 cycles at 1 mA cm^−2^ and 1 mAh cm^−2^. Additionally, Rate‐capability tests were performed at 0.5 and 5 mA cm^−2^ with 1 mAh cm^−2^ capacity (Figure 3d). When the current was returned to 0.5 mA cm^−2^, Li/ZnF_2_ recovered a low polarization (< 10 mV), whereas Bare Li retained > 100 mV, highlighting faster Li^+^ transport kinetics and enhanced interfacial stability. As shown in Figure 3e, Tafel analysis revealed a difference in charge transfer kinetics between the two electrodes. Bare Li exhibited an exchange current density of 0.133 mA cm^−2^, whereas Li/ZnF_2_ reached a substantially higher exchange current density of 0.318 mA cm^−2^. Within the Butler−Volmer equation, a higher exchange current density corresponds to faster interfacial charge‐transfer kinetics and enhanced Li^+^ transport across the electrode–electrolyte interface [12, 62].

The markedly improved kinetics of the Li/ZnF_2_ electrode arise from the SGI Li–Zn–F, which provides efficient Li^+^ diffusion pathways and facilitates rapid charge transfer within the electrode. As shown in Figure 3f, the voltage hysteresis, defined as the difference between the maximum and minimum voltages during each cycle, remained below 40 mV for Li/ZnF_2_ even after 500 cycles. In contrast, the Bare Li symmetric cell exhibited a rapid increase in polarization and short‐circuited after approximately 200 cycles, underscoring the superior interfacial stability of the Li/ZnF_2_ anode. Additional symmetric cell tests with varied current densities and areal capacities corroborate this stability. At 2 mA cm^−2^ and 1 mAh cm^−2^, Bare Li showed high overpotential within 100 h, while Li/ZnF_2_ maintained around 20 mV for 700 h (Figure S13). Under 2 mA cm^−2^ and 2 mAh cm^−2^ conditions, Bare Li reached 1000 mV after 200 h, whereas Li/ZnF_2_ sustained approximately 30 mV for 300 h (Figure S14). Even in a carbonate‐based electrolyte, Li/ZnF_2_ exhibited much more stable cycling than Bare Li at 1 mA cm^−2^ and 1 mAh cm^−2^ (Figure S15). Overall, the superior electrochemical performance of Li/ZnF_2_ symmetric cells arises from the SGI Li–Zn–F, which ensures stable Li nucleation, rapid ion transport, and robust interfacial integrity during long‐term cycling.

### 2.4 Interfacial Transformation of Li Anodes Upon Cycling

**FIGURE 4:**
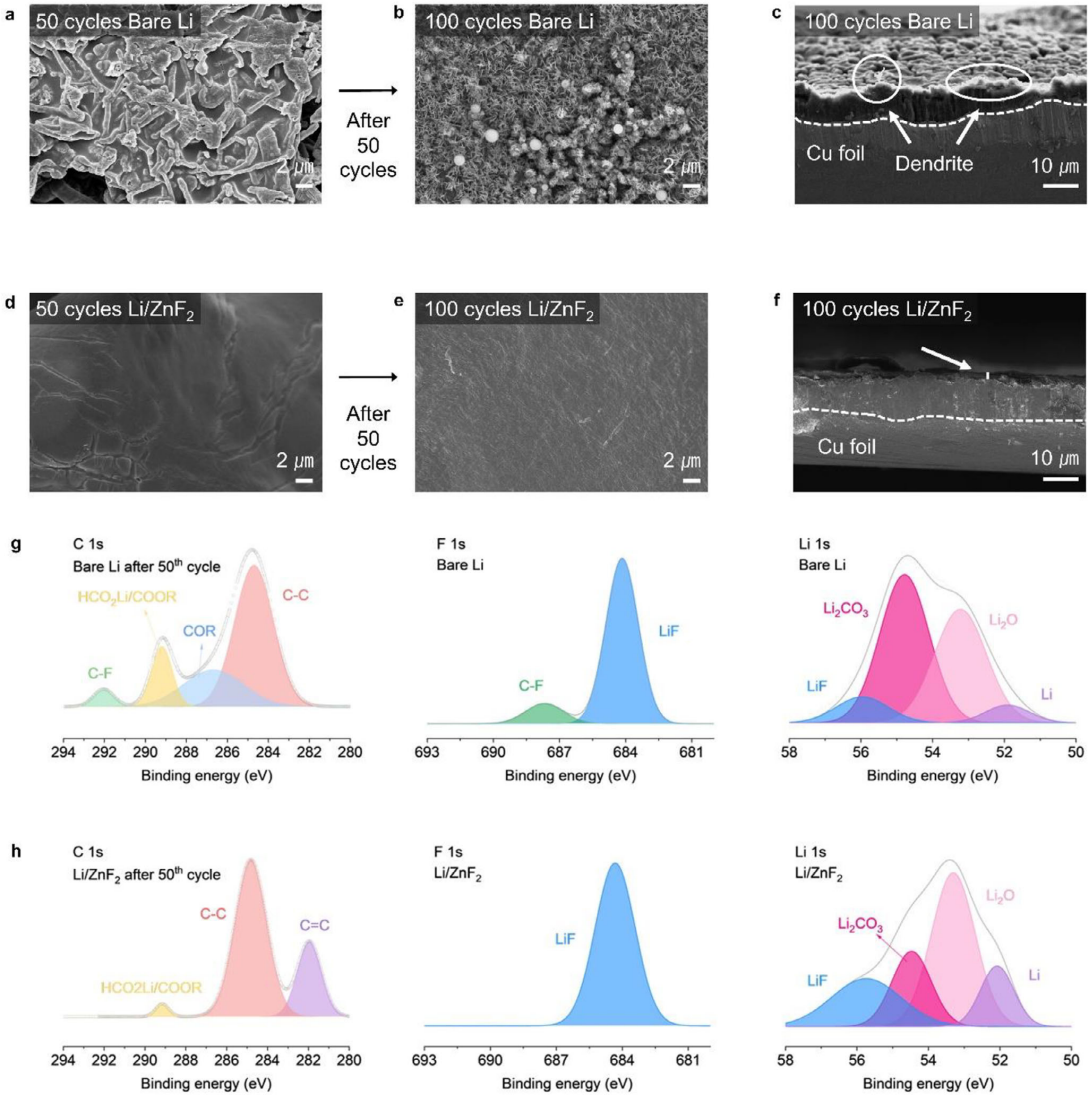
SEM images of the Bare Li at 1 mA cm^−2^ after (a) 50 cycles, (b) 100 cycles; (c) SEM images of the cross‐section of the Bare Li after 100 cycles. SEM image of the Li/ZnF_2_ at 1 mA cm^−2^/1mAh cm^−2^ after (d) 50 cycles, (e) 100 cycles; (f) SEM image of the cross‐section of the Li/ZnF_2_ 100 cycles. XPS spectra for (g) Bare Li C 1s, F 1s, and Li 1s; (h) Li/ZnF_2_ C 1s, F 1s, and Li 1s after 50 cycles.

The morphological evolution of Bare Li and Li/ZnF_2_ electrodes after cycling was examined to evaluate Li plating/stripping behavior. After 50 and 100 cycles, the surface morphology of Bare Li revealed extensive dendritic growth and severe degradation (Figure 4a,b). The surface after 50 cycles displayed a disordered network of lithium dendrites, and after 100 cycles, continuous electrolyte exposure induced significant side reactions. Cross‐sectional imaging further confirmed the presence of sharp, needle‐like dendritic structures protruding from the surface (Figure 4c). In contrast, Li/ZnF_2_ exhibited a dense and uniform morphology after 50 and 100 cycles (Figure 4d,e). The cross‐sectional image revealed a stable SEI layer and a dendrite‐free electrode surface, indicating that the lithiophilic SGI Li–Zn–F can stably accommodate lithium and facilitate uniform deposition during cycling (Figure 4f). In addition, after a single Li plating step at 1 mA cm^−2^ and 1 mAh cm^−2^, the Li/ZnF_2_ electrode exhibited a dense, low‐porosity surface compared with Bare Li (Figure S16). This morphology indicates that the SGI Li–Zn–F effectively promotes uniform lithium nucleation and deposition, consistent with the DFT predicted behavior shown in Figure 2.

To further probe interfacial stability, XPS depth profiling was performed on cycled Bare Li and Li/ZnF_2_ electrodes. As shown in Figure 4g,h, the Bare Li electrode exhibits C 1s XPS spectra featuring C═C (284.8 eV), COR (R: radical) (286.4 eV), HCO_2_Li/COOR (288.6 eV), and C─F_3_ (292.8 eV) [27, 63]. In contrast, the Li/ZnF_2_ electrode exhibited significantly lower amounts of HCO_2_Li/COOR formed on the surface due to irreversible side reactions, and peaks for COR and C─F_3_ were observed. Furthermore, as shown in the F 1s spectrum, Bare Li exhibited not only the LiF (685.0 eV) peak but also the byproduct peak C─F_3_ (688.8 eV) [64].

However, C─F_3_ was not observed on Li/ZnF_2_, and a higher LiF content was confirmed, which induces a uniform Li^+^ flux distribution and contributes to improved interface stability. In the Li 1s spectrum, the Li/ZnF_2_ electrode showed peaks for LiF, Li_2_CO_3_, Li_2_O, and Li. Notably, the amounts of Li_2_CO_3_ and Li_2_O formed by side reactions were significantly lower compared to Bare Li. Particularly, a larger quantity of the Li (52.0 eV) peak was observed than in Bare Li. These results demonstrated that SGI Li–Zn–F aids SEI formation, protecting the electrode from corrosion and side reactions caused by the electrolyte. Furthermore, its excellent ionic conductivity properties and uniform Li plating/stripping process enabled stable cell behavior. In contrast, the Bare Li electrode exhibited excessive irreversible side reactions during Li plating and stripping. Due to an uneven current distribution, C─F_3_ and carbonate‐related peaks were observed in the C 1s region. This indicates the formation of an unstable SEI on the surface.

EIS corroborated these findings (Figure S17). Before cycling, the interfacial resistance of Bare Li and Li/ZnF_2_ was 539.7 and 187.0 Ω, respectively; after 100 cycles, these values decreased to 166.0 and 6.3 Ω. The much lower resistance of Li/ZnF_2_ confirmed that the SGI Li–Zn–F facilitates rapid Li^+^ transport. The air and humidity stability of the electrodes was further evaluated (Figure S18). Bare Li oxidized and blackened within 24 h of air exposure and showed complete surface degradation after 72 h. In sharp contrast, Li/ZnF_2_ maintained a metallic luster and structural integrity without visible oxidation even after 72 h, confirming that the LiF‐rich upper layer significantly enhances resistance to air and moisture. This superior ambient stability originates from the chemically inert and dense LiF‐rich outer layer, which acts as an effective protective barrier against oxygen and moisture penetration. Such environmentally robust characteristics highlight the practical viability of the SGI Li–Zn–F architecture for lithium metal anodes under realistic handling and storage conditions.

### 2.5 Full‐Cell Performance of Li/ZnF_2_ Anode

**FIGURE 5:**
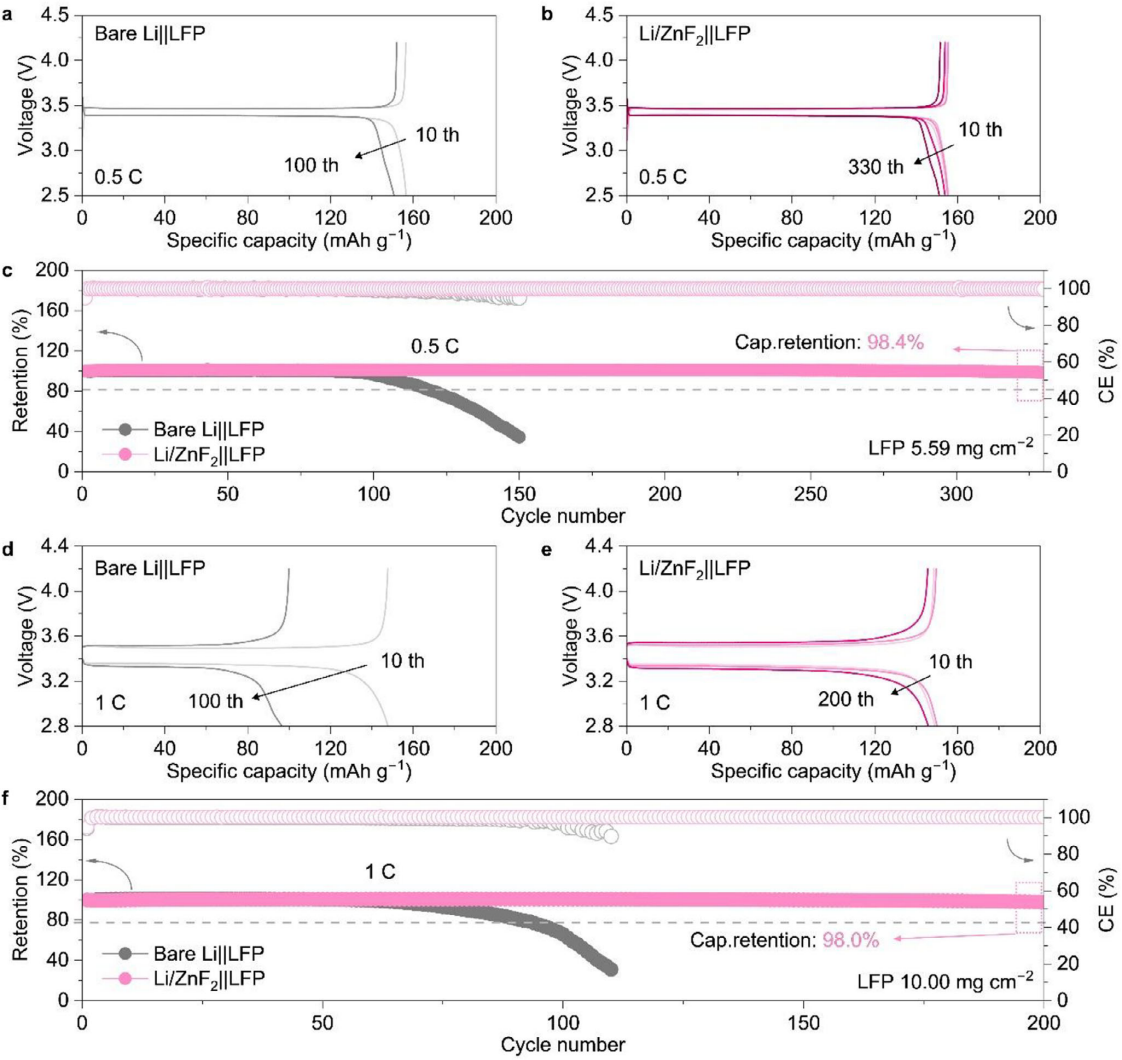
Corresponding voltage profiles of (a) the Bare Li||LFP and (b) Li/ZnF_2_||LFP and (c) cycling performance of Bare Li||LFP and Li/ZnF_2_||LFP at 0.5 C. Corresponding voltage profiles of (d) the Bare Li||LFP and (e) Li/ZnF_2_||LFP and (f) cycling performance of Bare Li||LFP and Li/ZnF_2_||LFP at 1 C.

Full cell evaluations were carried out to validate the high electrochemical performance of the Li/ZnF_2_ electrode. The LiFePO_4_ (LFP) cathode was prepared with a mass loading of 5.59 mg cm^−2^ and tested at 0.5 C (Figure 5a–c). The Bare Li||LFP cell exhibited a rapid capacity fade after 150 cycles, with the retention dropping 80% after 114 cycles. In contrast, the Li/ZnF_2_||LFP full cell maintained a high‐capacity retention of 98.4% over 330 cycles under identical conditions, demonstrating significantly enhanced cycling stability. At a higher rate of 1 C, the Bare Li||LFP cell showed severe degradation after 70 cycles, with the capacity declining to 98 mAh g^−1^ after 100 cycles (Figure 5d–f). Conversely, the Li/ZnF_2_||LFP cell delivered an initial discharge capacity of 156.2 mAh g^−1^ and retained 98.0% of its capacity after 200 cycles, underscoring its superior rate durability. Further confirmation was obtained using LCO with a mass loading of 10.00 mg cm^−2^ (Figure S19). At 0.5 C, the Li/ZnF_2_||LCO full cell achieved 75.2% capacity retention after 100 cycles, whereas the Bare Li||LCO counterpart exhibited rapid capacity decay after only 30 cycles, reflecting much poorer interfacial stability. Rate‐performance tests demonstrated that the Li/ZnF_2_||LFP full cell delivered reversible capacities of 160.4, 156.9, 152.9, 147.2, 142.4, and 134.5 mAh g^−1^ at current rates of 0.2, 0.5, 1.0, 2.0, 3.0, and 5.0 C, respectively higher than the corresponding values of 156.3, 152.9, 149.1, 143.7, 138.9, and 129.6 mAh g^−1^ obtained for the Bare Li||LFP full cell (Figure S20). These results collectively indicate faster Li^+^ transport kinetics and improved rate capability enabled by the Li/ZnF_2_ electrode. In addition, Li/ZnF_2_ anodes achieve remarkable capacity retention and cycling stability, attributed to the SGI Li–Zn–F, which facilitates uniform nucleation while maintaining interfacial.

### 2.6 Dendrite‐Suppression Mechanism Enabled by the Li–Zn–F Gradient Interphase

**FIGURE 6:**
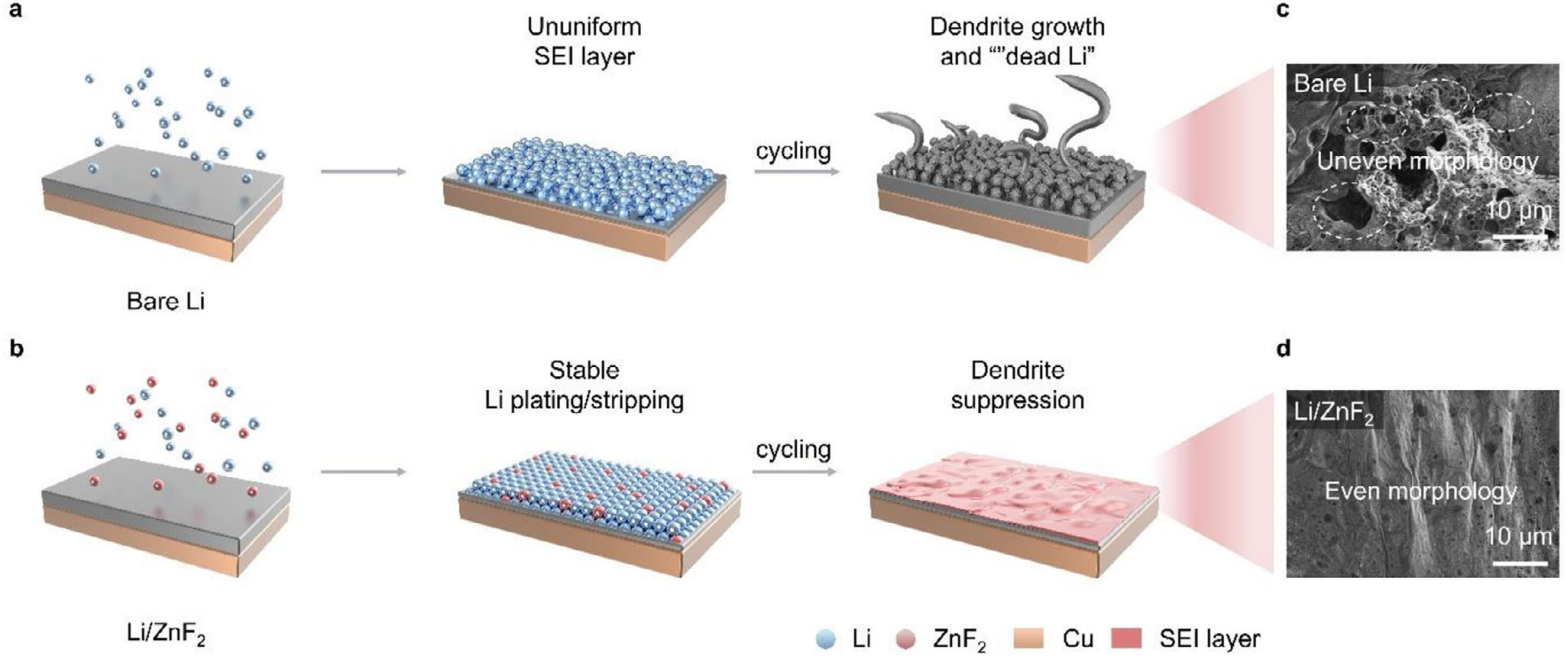
Schematic illustration of (a) the Bare Li and (b) Li/ZnF_2_ after Li plating/stripping. (c) The Bare Li and (d) Li/ZnF_2_ anode after 40 cycles in full cells with LFP mass loading of 4.16 mg cm^−2^.

The high electrochemical performance of the Li/ZnF_2_ electrode and its dendrite‐suppression mechanism are illustrated in Figure 6. For the Bare Li anode, lithium deposited on Cu foil exhibited nonuniform nucleation and growth due to the high nucleation overpotential, leading to uneven Li plating until stabilization was achieved. The resulting unstable SEI layer caused local current‐density fluctuations during plating/stripping, promoting dendritic growth and the accumulation of electrically isolated “dead Li,” which degraded cell performance (Figure 6a). In contrast, when molten Li was coated on the Li/ZnF_2_ substrate, the SGI Li–Zn–F spontaneously formed, consisting of a LiZn alloy layer at the bottom and a LiF layer at the top. This vertically graded configuration enables smooth Li^+^ transport through the highly conductive LiF layer adjacent to the electrolyte, while the lithiophilic LiZn alloy beneath provides abundant nucleation sites that facilitate uniform Li deposition on the Cu current collector. As a result, the SGI effectively suppresses dendritic propagation during cycling, ensuring the formation of a highly stable lithium metal interface.

To evaluate the degradation behavior and interfacial stability of the Li/ZnF_2_ anode, the LFP full cells were disassembled after 350 cycles at 1 C. As shown in Figure 6b, the Bare Li||LFP cell exhibited a severely damaged anode surface, characterized by a porous dendritic structure that developed during cycling. In contrast, the Li/ZnF_2_ anode maintained a flat and smooth surface even after prolonged cycling, indicating that the lithiophilic SGI Li–Zn–F effectively suppressed local current‐density hotspots and guided uniform Li deposition throughout repeated plating/stripping (Figure 6d). The preserved structural integrity after extended cycling further reflects the robustness of the LiF‐rich outer layer, which stabilizes the electrode/electrolyte interface during operation.

The LFP cathodes harvested from the Li/ZnF_2_||LFP and Bare Li||LFP cells were also examined by SEM after cycling. No significant morphological deformation or particle cracking was observed on either cathode, and no discernible differences were detected between the two samples. These observations suggest that anode degradation, rather than cathode deterioration, was the primary contributor to cell failure (Figure S21).

## 3 Conclusion

This study establishes generalized design principles for self‐forming gradient interphases (SGIs) that extend beyond the specific case of ZnF_2_. In such systems, Li–M alloy‐forming elements (M = Zn, Mg, Ag, Al, etc.) migrate toward the current collector, where they lower the nucleation overpotential and promote uniform Li deposition. Concurrently, halide‐based inorganic phases (LiF, LiCl, etc.) segregate toward the electrolyte interface, providing high ionic conductivity and robust interfacial protection. These role‐separated architectures arise spontaneously through melt‐driven reactions, without the need for vacuum deposition or complex microfabrication steps. Notably, the dip‐coating and melt‐reaction process demonstrated here is inherently compatible with scalable roll‐to‐roll electrode manufacturing and can be readily extended to large‐area lithium metal anodes. Therefore, the SGI concept represents not merely a materials innovation but a practical framework that bridges laboratory‐scale advances with industrial‐level lithium metal battery production.

## 4 Experimental Section

### 4.1 Preparation of the Li/ZnF_2_

Li/ZnF_2_ electrodes were fabricated in an Ar‐filled glove box. Li foil (Honjo Metal Co., Ltd.) and ZnF_2_ powder (≥ 99%, anhydrous, Thermofisher Scientific) were combined at a mass ratio of 20:1 and repeatedly rolled and folded to form a lithium composite metal. The composite was heated to 280°C on a hot plate using a stainless‐steel container and stirred thoroughly in the molten state. Cu foil was then coated by dip‐coating into the molten lithium composite to produce Li/ZnF_2_. Lithium reacted with ZnF_2_ to form an SGI Li–Zn–F that accelerates Li^+^ transport and induces uniform nucleation.

### 4.2 Preparation of the LFP and LCO

LFP cathodes were prepared by mixing active material, Super P, and polyvinylidene fluoride (PVDF) in N‐methyl pyrrolidinone (NMP) at mass ratios of 8:1:1 and 92:4:4, followed by coating onto Al foil and drying for 10 h. LCO cathodes were fabricated analogously at a 92:4:4 mass ratio. LFP mass loading was 4–17 mg cm^−2^ and LCO mass loading was 10.00 mg cm^−2^. The loading densities were 1.8 g cc^−3^ for LFP and 3.3 g cc^−3^ for LCO. LFP electrodes for coin cells were punched to 14 mm diameter; pouch‐cell electrodes were cut to 31  ×  50 mm^2^. LCO coin‐cell electrodes were 14 mm in diameter.

### 4.3 Materials Characterization

Surface and cross‐sectional morphologies were characterized by scanning electron microscopy (SEM, GEMINI500, 10 kV). The alloy structure of the SGI Li–Zn–F was characterized by x‐ray diffraction (XRD, Rigaku). The cross‐sectional morphology of the Li/ZnF_2_ electrode was examined using a focused ion beam (FIB, Scios, 5 kV, current: 0.2 nA) coupled with a SEM (GEMINI500, 10 kV). Elemental distribution was analyzed by energy‐dispersive x‐ray spectroscopy (EDS) attached to the SEM, and the corresponding images were qualitatively evaluated at an accelerating voltage of 15 kV. The chemical composition and interfacial states of the lithium‐metal phase and solid–electrolyte interphase were further examined using x‐ray photoelectron spectroscopy (XPS, AXIS Supra TM) with monochromatic Al Kα radiation (1486.6 eV).

### 4.4 Electrochemical Measurements

Tafel measurements were performed on a WonATech (WBCS3000L), scanned from −0.2 to 0.2 V at 1 mV s^−1^. Electrochemical impedance spectroscopy (EIS) was carried out on a WonATech (ZIVE SP1) from 200 kHz to 0.1 Hz.

### 4.5 Cell Assembly and Electrochemical Test

All symmetric cells (Bare Li and Li/ZnF_2_) were assembled in an Ar‐filled glove box using ether‐based electrolyte 1.0 M lithium bis(trifluoromethanesulphonyl) imide (LiTFSI) in 1,3‐dioxolane (DOL)/ 1,2‐dimethoxyethane (DME) (v/v = 1:1) with 2% LiNO_3_ (Soulbrain, South Korea) and, where specified, carbonate‐based electrolyte 1.1 m lithium hexafluorophosphate (LiPF_6_) in ethylene carbonate (EC)/ diethyl carbonate (DEC) (v/v = 1:1) with 10% fluoroethylene carbonate (FEC) (Soulbrain, South Korea). Celgard 2400 separators were used. Cycling was conducted on a WonATech (WBCS3000L). Li plating/stripping conditions employed current densities of 1, 2, and 4 mA cm^−2^ and areal capacities of 1, 2, 4, and 16 mAh cm^−2^; for rate tests, the areal capacity was fixed at 1 mAh cm^−2^, and current densities were 0.5, 1, 2, 3, and 5 mA cm^−2^.

All full cells (Bare Li and Li/ZnF_2_) were assembled in an Ar‐filled glove box with carbonate‐based electrolyte (1.1 m LiPF_6_ in EC:EMC, 1:1 v/v, with 10% FEC) and Celgard 2400 separators. Formation charge/discharge was conducted at 0.2 C for three cycles within 2.5 and 4.2 V for mass loading of LFP (5.59 mg cm^−2^) and within 2.8 and 4.2 V for mass loading of LFP (10.00 and 16.02 mg cm^−2^), after which cycling proceeded at 0.5 or 1 C as specified. For LCO (10.00 mg cm^−2^), formation charge/discharge was performed at 0.2 C within 3.0 and 4.3 V, followed by cycling at 0.5 C.

### 4.6 Theoretical Calculator

The detailed structural characteristics of the Li metal battery systems were investigated through density functional theory (DFT) calculations conducted with the Vienna Ab Initio Simulation Package (VASP) [65, 66, 67]. All calculations utilized the Perdew–Burke–Ernzerhof (PBE) formulation of the generalized gradient approximation (GGA) for the exchange–correlation functional [68], in combination with projector‐augmented‐wave (PAW) pseudopotentials [69], with the Monkhorst–Pack k‐point mesh [70] generated using an actual spacing of approximately 0.02 Å^−1^. A plane‐wave basis set with an energy cutoff of 500 eV was applied, and the convergence criteria were defined as 1.0 × 10^−5^ eV for total energy and 2.0 × 10^−2^ eV/Å for the atomic forces. In addition, the calculations incorporated the DFT‐D3 dispersion correction method [71] together with a dipole correction [72] applied along the *z*‐axis. The formation energy (*E_formation_*) was calculated as follows:

$$\;E_{formation}=E_{products}\;-E_{reactants}$$

where the *E_products_* and *E_reactants_* denote the total energy of the products and that of the reactants, respectively. To calculate the interface energy between materials, slab models consisting of three to five atomic layers were constructed, where the uppermost two layers were allowed to relax freely during the initial geometry optimization step. In this study, the Li(100) [73], Cu(111) [74], LiF(001) [75], and LiZn(110) [76] surfaces, which exhibit the lowest surface energies for each respective material, were employed. The interface energy (*E_interface_*), representing the adsorption energy at the Cu(100)|Li(100), Cu(100)|LiZn(110), and Cu(100)|LiF(100) interfaces, was calculated as follows:

$$\;E_{interface}=E_{a|b}\;-E_{a}-E_{b}$$

where the *E*_*a*|*b*_, *E_a_*, and *E_b_* denote the total energy of the entire structure and the energies of adsorption structures a and b, which correspond to the configurations before adsorption, respectively. The Li adsorption energies ($E_{Li\_adsoption}$) on Li(100), LiZn(110), and LiF(100) surfaces were calculated as follows:

$$\;E_{Li\_adsoption}=E_{slab+Li}\;-E_{slab}-E_{Li}$$

where the *E*_*slab* + *Li*_, *E_slab_*, and *E_Li_* denote the total energy of the slab with a single Li atom adsorbed on the Li(100), LiZn(110), or LiF(100) surface, the total energy of the slab, and the energy of an isolated Li atom, respectively. The energy barrier for lithium migration between designated sites was determined using the climbing image nudged elastic band (CI‐NEB) [77] technique with five intermediate images.

## Conflicts of Interest

The authors declare no conflicts of interest.

## Supporting information




**Supporting File**: smll74228‐sup‐0001‐SuppMat.docx.

## Data Availability

The data that support the findings of this study are available from the corresponding author upon reasonable request.
