# Supplementary material for: Regulating Lithium Plating Behavior in Lithium‐Metal Batteries via Molten‐Lithium Processing With Inorganic Additives
Source: Small. 2026 Jun 18;22(44):e74228. doi: 10.1002/smll.74228 (PMC13450048; doi:10.1002/smll.74228)
Supplement: Supplementary file 1 [file SMLL-22-e74228-s001.docx]

Supporting Information

**Regulating Lithium Plating Behavior in Lithium-Metal Batteries via Molten-Lithium Processing with Inorganic Additives**

*Chaerim Kim* *^†^, Jihyeon Kang* *^†^, jinyeong Choi, Chaerin Jung, Hyeongbeen Kim, Pilgun Oh, Jin Hong Lee***, Sung Hyun Kwon***, Minjoon Park**

**Figure S1.** SEM image of ZnF_2_ powder and EDS mapping image of (b) Zn and (c) F.

**Figure S2.** Fabrication of Li/ZnF_2_ electrode.

**Figure S3.** XRD pattern of Cu foil.

**Figure S4.** XRD pattern of the Bare Li.

**Figure S5.** (a-c) Cross-section SEM image and (d-i) the corresponding EDS mapping of the interface between the Li/ZnF_2_ layer and Cu substrate.

**Figure S6.** Formation energies of the (a) LiF and Li (b) LiF and LiZn.

**Figure S7.** Different possible adsorption sites on the Li (100): (a) Top; (b) Bridge; (c) Hollow.

**Figure S8.** Different possible adsorption sites on the LiZn (110): (a) Li-top; (b) Zn-top; (c) Zn-Zn bridge; (d) Li-Li bridge; (e) Zn-Zn-Zn-Li hollow; (f) Li-Li-Li-Zn hollow; (g) Zn-Zn-Li hollow; (h) Li-Li-Zn hollow; (i) Li-Zn bridge.

**Figure S9.** Different possible adsorption sites on the LiF (100): (a) Li-Top; (b) F-Top; (c) Bridge; (d) Hollow.

**Figure S10.** Cycling performance of Bare Li and Li/ZnF_2_ symmetric cells with different mass ratios (10%, 20%, and 30%) at 1 mA cm^−2^ and 1 mAh cm^−2^.

**Figure S11.** Nyquist plots of the Li/ZnF_2_ symmetric cells with different mass ratios (10%, 20%, and 30%).

**Figure S12.** Galvanostatic plating and stripping voltage profiles at 4 mA cm^−2^ and 16 mAh cm^-2^ of the symmetric cells with Bare Li and Li/ZnF_2_.

**Figure S13.** Galvanostatic plating and stripping voltage profiles at 2 mA cm^−2^ and 1 mAh cm^-2^ of the symmetric cells with Bare Li and Li/ZnF_2_.

**Figure S14.** Galvanostatic plating and stripping voltage profiles at 2 mA cm^−2^ and 4 mAh cm^-2^ of the symmetric cells with Bare Li and Li/ZnF_2_.

**Figure S15.** Galvanostatic plating and stripping voltage profiles at 1 mA cm^−2^ of the symmetric cells with Bare Li and Li/ZnF_2_ using 1.0 M LiPF_6_ in EC:DEC (1:1) with 10% FEC.

**Figure S16.** Top-view SEM images of (a) Bare Li and (b)Li/ZnF_2_ electrodes after Li deposition at 1 mAh cm^-2^.

**Figure S17.** Nyquist plots of the Li/ZnF_2_ symmetric cells (a) before cycling and (b) after 100 cycles at 1 mA cm^-2^ and 1 mAh cm^-2^.

**Figure S18.** The surface change of Bare Li electrode and Li/ZnF_2_ exposed in a controlled environment with relative humidity (RH) and air.

**Figure S19.** (a) Cycling performance of the Bare Li||LCO and Li/ZnF_2_||LCO with LCO the mass loading of 10 mg cm^−2^ at 0.5 C and voltage profiles of the (b) Bare Li||LCO and (c) Li/ZnF_2_||LCO

**Figure S20.** (a) Rate capability of the Bare Li and Li/ZnF_2_ with LFP the mass loading of 5.00 mg cm^−2^ at 0.5 C and voltage profiles of the (b) Bare Li||LFP and (c) Li/ZnF_2_||LFP at the different rates from 0.2 to 5 C.

**Figure S21.** (a), (b) Bare Li||LFP and (c), (d) Li/ZnF_2_||LFP after the 350th cycle in the LFP-based full cells.

| LMB  (year) | Reference | Cathode | Mass loading  (mg cm^–2^) | C-rate  (C) | Cycle (s) |
| --- | --- | --- | --- | --- | --- |
| **This work** | Li/ZnF_2_ | LFP | 5.59 | 0.5 | 330 |
| Lu et al. (2024) | [1] | LFP | 8.7 | 0.5 | 220 |
| Wu et al (2023) | [2] | NCM811 | 3 | 0.2 | 100 |
| Sun et al. (2021) | [3] | LCO | 12.5 | 0.5 | 130 |
| Han et al. (2024) | [4] | NCM811 | 14.5 | 0.5 | 80 |

**Table S1.** Performance comparison of the LMB with different cathode mass loading.

**References**

[1]	J. Cao, Y. Shi, A. Gao, et al., “Hierarchical Li electrochemistry using alloy-type anode for high-energy-density Li metal batteries,” *Nature communications* 15, no. 1 (2024): 1354.

[2]	J. Yang, C. Chen, K. Kashif, et al., “Melting lithium alloying to improve the affinity of Cu foil for ultra-thin lithium metal anode,” *Journal of Colloid and Interface Science* 630, no. (2023): 901-908.

[3]	J. Du, W. Wang, M. Wan, et al., “Doctor‐blade casting fabrication of ultrathin Li metal electrode for high‐energy‐density batteries,” *Advanced Energy Materials* 11, no. 45 (2021): 2102259.

[4]	Y. Chen, Q. Zhang, K. Wang, et al., “Realizing Ultrathin LiSn Alloy Anode by Tuning the Wettability of Molten Li for High-Energy-Density Batteries,” *ACS Applied Materials & Interfaces* 16, no. 22 (2024): 28570-28577.
